# Non-small cell lung cancer with *MET* amplification: review of epidemiology, associated disease characteristics, testing procedures, burden, and treatments

**DOI:** 10.3389/fonc.2023.1241402

**Published:** 2024-01-11

**Authors:** Mo Yang, Erin Mandal, Frank X. Liu, Richard M. O’Hara, Beth Lesher, Rachel E. Sanborn

**Affiliations:** ^1^ North America Evidence and Value Development, North America Medical Affairs, EMD Serono, Inc., Rockland, MA, United States, an affiliate of Merck KGaA; ^2^ Evidence and Access, OPEN Health, Parsippany, NJ, United States; ^3^ Earle A. Chiles Research Institute, Providence Cancer Institute, Portland, OR, United States

**Keywords:** non-small cell lung carcinoma, epithelial-mesenchymal transition, systematic review, epidemiology, treatment outcome

## Abstract

**Introduction:**

Mesenchymal-epidermal transition factor gene amplification (*MET*amp) is being investigated as a therapeutic target in advanced non-small cell lung cancer (NSCLC). We reviewed the epidemiology and disease characteristics associated with primary and secondary *MET*amp, as well as the testing procedures used to identify *MET*amp, in advanced NSCLC. Economic and humanistic burdens, and the practice patterns and treatments under investigation for *MET*amp were also examined.

**Methods:**

Embase and Medline (via ProQuest), ClinicalTrials.gov, and Cochrane Controlled Register of Trials (2015–2022) were systematically searched. Conference abstracts were searched via Embase and conference proceedings websites (2020–2022). The review focused on evidence from the United States; global evidence was included for identified evidence gaps.

**Results:**

The median rate of primary *MET*amp in NSCLC across the references was 4.8% (n=4 studies) and of secondary *MET*amp (epidermal growth factor receptor [*EGFR*]-mutant NSCLC) was 15% (n=10). Next-generation sequencing (NGS; n=12) and/or fluorescence *in situ* hybridization (FISH; n=11) were most frequently used in real-world studies and FISH testing most frequently used in clinical trials (n=9/10). *MET*amp definitions varied among clinical trials using ISH/FISH testing (MET to chromosome 7 centromere ratio of ≥1.8 to ≥3.0; or gene copy number [GCN] ≥5 to ≥10) and among trials using NGS (tissue testing: GCN ≥6; liquid biopsy: *MET* copy number ≥2.1 to >5). Limited to no data were identified on the economic and humanistic burdens, and real-world treatment of *MET*amp NSCLC. Promising preliminary results from trials enrolling patients with *EGFR*-mutated, *MET*amp advanced NSCLC progressing on an EGFR-tyrosine kinase inhibitor (TKI) were observed with MET-TKIs (i.e., tepotinib, savolitinib, and capmatinib) in combination with EGFR-TKIs (i.e., gefitinib and osimertinib). For metastatic NSCLC and high-level *MET*amp, monotherapy with capmatinib, crizotinib, and tepotinib are recommended in the 2022 published NSCLC NCCN Guidelines.

**Conclusion:**

Primary *MET*amp occurs in approximately 5% of NSCLC cases, and secondary *MET*amp in approximately 15% of cases previously treated with an EGFR inhibitor. Variability in testing methods (including ISH/FISH and NGS) and definitions were observed. Several treatments are promising in treating *MET*amp NSCLC. Additional studies evaluating the clinical, economic, and humanistic burdens are needed.

## 1 Introduction

Non-small cell lung cancer (NSCLC) is a heterogenous disease that is frequently diagnosed at an advanced stage due to variability of signs and symptoms at diagnosis (1, 2). The treatment of NSCLC is becoming more individualized as broad molecular testing for actionable driver alterations has transformed the diagnosis and treatment of advanced NSCLC (3). As of 2023, both national and international guidelines recommend molecular testing to help guide treatment decisions in advanced NSCLC (4–6).

Alterations in the transmembrane tyrosine kinase mesenchymal-epidermal transition factor (MET) receptor such as *MET* gene amplification (*MET*amp) have been identified as actionable drivers in NSCLC (7). *MET*amp in NSCLC may be a primary oncogenic driver, or a secondary driver which arises during or following treatment and commonly develops as an adaptive resistance mechanism to epidermal growth factor receptor (EGFR)-tyrosine kinase inhibitor (TKI) treatment (7, 8). Although EGFR-TKIs have significantly improved outcomes in patients with *EGFR*-mutant advanced NSCLC, the development of *MET*amp-driven resistance to EGFR-TKIs is a key obstacle to long-term disease control (4, 9). In patients with secondary *MET*amp following treatment with an EGFR-TKI, concomitant inhibition of both *EGFR* and *MET* is thought to overcome resistance to EGFR inhibitors due to *MET*amp (9).

This targeted review was undertaken to better understand the burden of *MET*amp NSCLC in the United States (US) and to identify the available evidence on the epidemiology and disease characteristics associated with primary and secondary *MET*amp. Other outcomes evaluated included evidence on *MET*amp testing procedures, the economic and humanistic burdens of *MET*amp NSCLC, and practice patterns and treatments under investigation for *MET*amp advanced NSCLC.

## 2 Methods

A systematic search of studies published as journal articles or conference abstracts written in the English language was performed on June 14, 2022 using predefined search terms in Embase and Medline (via ProQuest; **Supplementary Table 1**). Journal articles were searched from January 1, 2015 onwards, and conference abstracts were searched from January 1, 2020 onwards. Abstracts from the following 2022 conferences that were not yet indexed in Embase by June 14, 2022 were also searched: the American Association for Cancer Research, Academy of Managed Care Pharmacy, American Society of Clinical Oncology, International Society for Pharmacoeconomics and Outcomes Research, and NCCN. Searches using predefined search terms in ClinicalTrials.gov (**Supplementary Table 2**) and the Cochrane Controlled Register of Trials (**Supplementary Table 3**) were performed to identify completed and ongoing clinical trials; searches were performed on June 14, 2022.

The utilized search strategies were global in scope and included terms for NSCLC, *MET*amp, and outcomes of interest. Whereas the focus of this literature review was US-based evidence, global, non-US-based evidence was included when gaps in US-based evidence for outcomes of interest were identified. Publications containing information on the following topics for either primary or secondary *MET*amp were selected for inclusion (**Table 1**): US-based epidemiology and global evidence for disease characteristics, genomic testing procedures, economic and humanistic burden, real-world treatment patterns, ongoing clinical trials, and clinical trial outcomes. Real-world prospective or retrospective studies, clinical trials, and US-based guidelines were included.

**Table 1:** ICOS selection criteria.

	Inclusion criteria	Exclusion criteria
**Population**	• Patients with advanced NSCLC and *MET*amp including both primary and secondary *MET*amp; the primary focus of the literature review is on secondary *MET*amp in patients with NSCLC who progressed on a TKI	• Patients with NSCLC harboring other genomic mutations without *MET*amp (e.g., *KRAS*, *ROS1*)
**Interventions**	• Any intervention	None
**Outcomes**	• Epidemiological outcomes (US-based prevalence, US-based incidence) • Rates of co-existence of *MET*amp with other actionable driver mutations • Patient and/or social status characteristics associated with *MET*amp NSCLC • Diagnosis and testing outcomes (including testing strategies, tumor vs liquid, time of testing, time interval between testing and results, and reporting) for *MET*amp NSCLC • Economic burden (including direct costs, indirect costs, and healthcare resource use) of advanced *MET*amp NSCLC • Patient-reported outcomes for patients with *MET*amp NSCLC • Real-world treatment patterns and outcomes (survival, progression-free survival, response rates, duration of treatment, treatment discontinuation due to adverse events, patient-reported outcomes) in patients with advanced *MET*amp NSCLC • Clinical trial outcomes (survival, progression-free survival, response rates, duration of treatment, treatment discontinuation, patient-reported outcomes), and any ongoing clinical trials in patients with advanced *MET*amp NSCLC • US-based treatment guidelines with recommendations for advanced *MET*amp NSCLC	None
**Study design**	• Real-world prospective or retrospective studies such as: chart reviews, database analyses, product or disease registries • Clinical trials • Guidelines • Systematic literature reviews^a^	• Review articles
**Other**	• English language papers only • Journal articles or conference abstracts • Journal articles published from 2015 to present • Conference articles published from 2020 to present	• Non-English papers (even if abstract is available in English)

a Systematic literature reviews were excluded but were used for identification of primary studies.

*KRAS*, Kristin rat sarcoma viral oncogene homolog; *MET*amp, mesenchymal-epithelial transition factor amplification; NSCLC, non-small cell lung cancer; PICOS, patient/population, intervention, comparison and outcomes; *ROS1*, c-ros oncogene 1; TKI, tyrosine kinase inhibitor.

An independent researcher completed title, abstract and full text screening, identified studies to be included based on the Population, Intervention, Comparison, Outcomes, Study Design (PICOS) selection criteria (**Table 1**), and extracted data. Another independent researcher performed standard quality checks on approximately 10% of randomly selected references during the abstract and full text screening to verify results. Results are descriptive in nature. No formal statistical analyses or comparisons among identified studies were made.

## 3 Results

**Figure 1:**
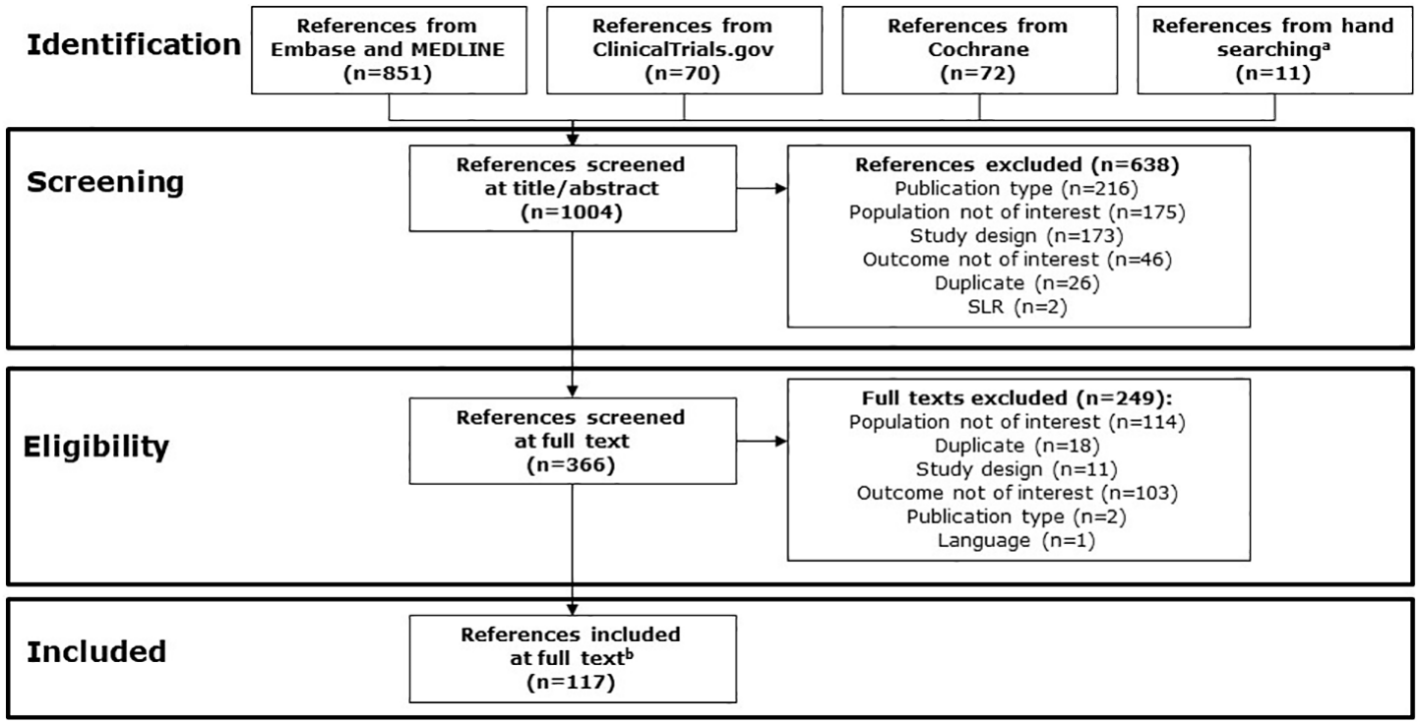
PRISMA study identification flow chart. ^a^ Abstracts from the following conferences in 2022 were searched via conference proceedings websites: AACR, AMCP, ASCO, ISPOR, and NCCN. References from relevant SLRs were reviewed. ^b^ Includes 79 publications from ProQuest and searching conferences, and 38 references from ClinicalTrials.gov. AACR, American Association for Cancer Research; AMCP, Academy of Managed Care Pharmacy; ASCO, American Society of Clinical Oncology; ISPOR, International Society for Pharmacoeconomics and Outcomes Research; NCCN, National Comprehensive Cancer Network; PRISMA, Preferred Reporting Items for Systematic reviews and Meta-Analyses; SLR, systematic literature review.

A total of 1,004 references were screened; of these, 117 references met the inclusion criteria, including 79 publications from ProQuest and searched conference abstracts, and 38 webpage references from ClinicalTrials.gov (**Figure 1**). Of the 117 references meeting inclusion criteria, references commonly provided information on more than one topic of interest.

### 3.1 Epidemiology and disease characteristics

A total of 13 publications providing information on the epidemiology of primary or secondary *MET*amp in the US were identified. The median incidence of primary *MET*amp in all NSCLC in the US was 4.8% (range: 2.0–5.8%), based on four studies including 3,379 patients (10–13). In these studies, primary *MET*amp was defined as *MET*amp at NSCLC diagnosis and/or before receipt of targeted therapies; *MET*amp was identified either by a single test or by a testing panel that included *MET*amp and other driver alterations (10–13). The median incidence of secondary (acquired) *MET*amp in *EGFR*-mutant NSCLC previously treated with an EGFR-TKI was 15% (range: 2.9–66.7%), based on a total of 10 studies including 389 patients (13–22).

A total of 29 publications providing information on disease characteristics of *MET*amp NSCLC were identified (23–51). A summary of results from 16 US- or non-US based studies that evaluated demographic and clinical characteristics of patients with NSCLC with or without *MET*amp is found in **Supplementary Table 4** (24–27, 29, 32, 33, 35–37, 44–47, 50, 51). Results from the identified studies show that the following characteristics were consistently considered not associated with *MET*amp (either primary, secondary, or not specified): age (11 studies), sex (11 studies), and NSCLC sub-type (adenocarcinoma vs non-adenocarcinoma, squamous cell carcinoma, or other; 6 studies) (24, 26, 27, 32, 33, 36, 44, 45, 47, 50, 51). Results from two studies demonstrated a significant statistical association between *MET*amp and a higher proportion of programmed cell death protein 1 ligand (PD-L1) expression (25, 35). Overall, most studies (8/10) found no statistical association between *MET*amp and a positive smoking history (24, 26, 27, 32, 33, 44, 45, 47, 50, 51). Among two studies that evaluated patients with *EGFR-*mutant NSCLC who experienced progression on an EGFR-TKI, one study found that history of smoking was associated with a high probability of secondary *MET*amp (p=0.011), and one study found no association between smoking status and presence of *MET*amp (p=0.45) (24, 27). No studies specifically looked at the association between demographics and clinical characteristics in patients with primary or secondary *MET*amp.

Results for patients with stage I–III NSCLC (n=170) show that patients with *MET*amp versus no *MET*amp had a significantly shorter time to development of distant metastasis (11.6 vs 43.8 months; p=0.004), and results of a multivariate analysis confirmed that *MET*amp was highly associated with earlier progression to distant metastases (hazard ratio: 4.86; 95% CI: 1.85, 12.75; p=0.001) (36). All patients in this study received standard of care therapy per their disease stage at diagnosis, such as surgery, chemotherapy, radiation therapy, or EGFR inhibitor therapy when applicable; rates of therapy and primary versus secondary *MET*amp were not reported. A second study found that among primary lung adenocarcinoma and metastatic lung adenocarcinoma tumor samples, the identification of *MET*amp was significantly higher in the metastatic than primary tumor samples (p<0.001) (46). Evidence regarding the association between brain metastases and *MET*amp differed based on the enrolled populations. Results from two different studies evaluating NSCLC tumor samples found that *MET*amp was identified in a significantly higher number of NSCLC brain metastases samples (including lung adenocarcinoma brain metastases) compared with samples from primary tumors (29, 37). Results from one study evaluating patients with advanced NSCLC with *EGFR* mutations found that rates of brain metastases did not significantly differ among patients with primary *MET*amp versus no *MET*amp (45).

Interpretation of survival results for patients with *MET*amp versus no *MET*amp is limited due to differences in follow-up times, treatments, and patient populations. Among all studies identified, no statistical association between *MET*amp and overall survival for patients with advanced NSCLC was found in six studies (24, 26, 27, 35, 44, 51), and significantly worse overall survival among patients with *MET*amp NSCLC versus non-*MET*amp NSCLC was found in three studies (36, 45, 47). Results from two studies evaluating patients with secondary *MET*amp who had *EGFR-*mutant NSCLC and progression on an EGFR-TKI found that there was no significant difference in overall survival from the time of initiation of EGFR-TKI therapy according to *MET*amp status (24, 27). Median progression-free survival from the most recent EGFR-TKI treatment was significantly shorter among patients with *MET*amp versus no *MET*amp, based on a multivariate analysis in one study (hazard ratio, 0.898; 95% CI: 0.835, 0.965; p=0.004) (24).

### 3.2 Testing

A total of 39 publications providing testing data for *MET*amp (including two US-based guidelines providing testing recommendations for *MET*amp NSCLC) were identified. The US-based guidelines included the NCCN Guidelines and the joint guideline by the College of American Pathologists (CAP), the International Association for the Study of Lung Cancer (IASLC), and the Association for Molecular Pathology (AMP) (4, 5).

The NSCLC NCCN Guidelines consider high-level *MET*amp to be an emerging biomarker, which should optimally be identified when broad molecular profiling is performed for the following actionable driver mutations: *EGFR*, anaplastic lymphoma kinase (*ALK*), Kristin rat sarcoma virus (*KRAS*), c-Ros oncogene 1 (*ROS1*), v-Raf murine sarcoma viral oncogene homolog B (*BRAF*), neurotrophic tyrosine receptor kinase (*NTRK1/2/3), MET* exon 14 skipping, RET proto-oncogene (*RET*), and human epidermal growth factor receptor 2 (*ERBB2 [HER2])* (4). The CAP-IASLC-AMP guidelines (published in 2018) recommend that it is appropriate to include *MET* alterations as part of larger testing panels performed either initially or when routine *EGFR*, *ALK*, and *ROS1* testing are negative (5). Molecular testing for *MET* alterations including *MET* exon 14 mutation and *MET*amp is not indicated as a routine stand-alone assay outside the context of a clinical trial per the CAP-IASLC-AMP guidelines.

The definition of high-level *MET*amp is evolving and may differ according to the assay used for testing (4). Fluorescence *in situ* hybridization (FISH) is generally considered the gold standard to evaluate *MET* gene copy number (52, 53). Tissue-based next-generation sequencing (NGS) can also be used to simultaneously test for *MET*amp and other actionable biomarkers (4). There are no clinically defined cut-off values for NGS. While the NCCN Guidelines recognize that the definition of high-level *MET*amp is not established, a copy number greater than 10 is considered consistent with characterizing a result as high-level *MET*amp (4). Of 10 identified clinical trials providing definitions for *MET*amp, FISH testing was the most commonly used testing strategy (nine studies) (30, 54–62). The definition for *MET*amp in clinical trials varied and ranged from a *MET* to chromosome 7 centromere (*MET*/CEP7) ratio of ≥1.8 to ≥3.0 (**Table 2**) (30, 54–62).

**Table 2:** Definitions of *MET*amp using FISH or NGS in clinical trials.

Reference	ClinicalTrials.gov identification number	*MET*amp definition
ISH/FISH (tumor tissue) *MET*amp definitions		
Dagogo-Jack 2021 (54)	NCT02750215	*MET/CEP7* ratio ≥1.8
Camidge 2021 (30)	NCT00585195	*MET/CEP7* ratio ≥1.8
Landi 2019 (55)	NCT02499614	*MET/CEP7* ratio >2.2
Wu 2020 (61)	NCT01982955	*MET/CEP7* ratio ≥2, or GCN ≥5
Wu 2018 (56)	NCT01610336	*MET/CEP7* ratio ≥2.0, or GCN ≥5
Sequist 2020 (57)	NCT02143466	*MET/CEP7* ratio ≥2, or GCN ≥5
Angevin 2017 (58)	NCT01391533	*MET/CEP7* ratio ≥2 and ≥10% of cells with >4 *MET* gene copies
Camidge 2020 (59)	NCT02648724	*MET/CEP7* ratio >2.2 updated to ≥3.0
Wolf 2020 (60)	NCT02414139	GCN ≥10 (cohorts with GCN <4, 4 or 5, and 6–9 were closed for futility)
NGS (tumor tissue) *MET*amp definitions		
Dagogo-Jack 2021 (54)	NCT02750215	GCN ≥6
Camidge 2021 (30)	NCT00585195	GCN ≥6
Sequist 2020 (57)	NCT02143466	≥20% tumor cells, coverage of ≥200×sequencing depth, and ≥5 copies of *MET* over tumor ploidy
NGS (liquid biopsy) *MET*amp definitions		
Le 2022 (62)	NCT02864992	*MET* copy number ≥2.5
Dagogo-Jack 2021 (54)	NCT02750215	*MET* copy number ≥2.1
Camidge 2020 (59)	NCT02648724	*MET* copy number >5

(F)ISH, (fluorescence) in situ hybridization; GCN, gene copy number; MET, mesenchymal-epithelial transition factor; *MET*amp, mesenchymal-epithelial transition factor amplification; *MET/CEP7, MET* to chromosome 7 centromere ratio; NGS, next-generation sequencing.

Most real-world studies that included data from the US (20 publications from the US and three publications from multi-country assessments including the US) used NGS (12 studies) and/or FISH (11 studies) to test for *MET*amp; among these studies, tissue samples were used in 13 studies, tissue or liquid samples in seven studies, liquid samples in one study, and no sample information was provided for two studies (10–14, 17, 18, 20, 25, 28, 36–38, 40, 49, 63–70). There are limited real-world data comparing the reliability of using NGS versus FISH to identify patients with *MET*amp (52, 53). Results from a study in Germany suggest that there is higher concordance between NGS and FISH when considering highly amplified *MET* (gene copy number >10); however, NGS may be less able to detect cases harboring lower levels of expression (i.e., low or intermediate *MET*amp) (52).

No studies evaluating the economic impact of specifically testing for *MET*amp were identified in the literature review; however, two studies evaluating the economic impact of using NGS versus single gene testing strategies to identify actionable genomic alterations among patients with advanced NSCLC in the US were identified (71, 72). Both studies demonstrated cost savings with NGS from both a Medicare and US commercial payer’s perspective when testing for actionable genomic alterations included in clinical guideline recommendations (*EGFR*, *ALK*, *ROS1*, *BRAF*, *KRAS*, *MET*, *HER2*, *RET*, and *NTRK1*) compared with single gene testing strategies (71, 72). NGS was associated with a faster mean time to appropriate targeted therapy initiation (2 weeks) compared with other testing strategies, including single gene sequential testing (8–9 weeks) or hotspot panel testing (3 weeks) (71).

### 3.3 Economic burden and patient-reported outcomes

No information on economic or humanistic burdens in *MET*amp NSCLC was identified in the literature.

### 3.4 Real-world treatment patterns and outcomes data

A total of 18 publications providing information on the real-world treatment patterns and outcomes data for *MET*amp NSCLC were identified. Results from several studies indicated that patients with *EGFR-*mutant NSCLC who received a variety of first-, second-, or third-generation EGFR-TKIs may have detection of secondary *MET*amp following progression on EGFR-TKI treatment (13, 24, 27, 42, 73, 74). Evidence from real-world studies suggests that there is no association between the type of EGFR-TKI and risk of acquiring *MET*amp (13, 24, 42).

There is a lack of US-based evidence on treatments used in real-world settings following diagnosis of *MET*amp NSCLC. The majority of references evaluating real-world treatment patterns among patients who have secondary *MET*amp NSCLC were from China and assessed crizotinib alone or in combination with EGFR-TKIs (such as gefitinib, erlotinib, osimertinib, and icotinib; **Supplementary Table 5**) (27, 39, 42, 73–75).

### 3.5 Investigational treatments in *MET*amp NSCLC

Inhibition of MET signaling is being investigated as a promising therapeutic strategy in patients with *MET*amp NSCLC; a total of 16 publications providing information on clinical trial evidence in *MET*amp NSCLC were identified. Several agents alone or in combination with EGFR-TKIs are currently under investigation for the treatment of *MET*amp NSCLC, including small molecule MET receptor inhibitors (e.g., crizotinib (30, 55), savolitinib (57, 76, 77), tepotinib (61, 62, 78, 79), and capmatinib (56, 60)), monoclonal or bispecific antibodies that can block MET activity (e.g., amivantamab (80)), and an anti-MET antibody–drug conjugate (e.g., telisotuzumab vedotin (81)).

Thirty-eight Phase I–III trials evaluating treatments for *MET*amp NSCLC and registered on ClinicalTrials.gov were identified as of June 14, 2022 (80, 82–118). Many identified trials included patients with several different *MET* alterations (including *MET* exon 14 skipping, MET overexpression, and *MET*amp), and some trials included patients with a variety of different actionable genomic alterations. Only results from trials that focus exclusively on *MET*amp or include a subgroup of patients specifically with *MET*amp are included in this review. Studies that only presented combined results for patients with *MET*amp and another *MET* alteration are not described in this review.

#### 3.5.1 MET tyrosine kinase inhibitors

There are two types of MET-TKIs, including type I MET-TKIs that bind to an active form of MET (e.g., tepotinib, capmatinib, crizotinib, savolitinib, and bozitinib), and type II MET-TKIs that bind to an inactive form of MET (e.g., cabozantinib and glesatinib). The majority of MET-TKIs are orally administered (e.g., tepotinib, capmatinib, savolitinib, crizotinib) (30, 55–57, 60–62, 76, 78, 79).

Published results identified at the time of this literature review from clinical trials evaluating MET-TKIs are summarized in the following sections and in **Table 3**.

**Table 3:** Overview of clinical trial results for MET-TKIs under evaluation in *MET*amp advanced NSCLC.

Reference	Patients required to have previously progressed on an EGFR-TKI?	Treatment (n)	Trial phase	ORR (CR or PR)	Median PFS (95% CI), months
Wu 2020 (61) Liam 2022 (78)	Yes	Tepotinib + gefitinib (12)	Phase Ib/II	67%	16.6 (8.3, 22.1)
Le 2022 (62)	No	Tepotinib (24)	Phase II	42%	4.2 (1.4, 15.6)
Wu 2018 (56)	Yes	Capmatinib + gefitinib (36)	Phase Ib/II	47%	5.49 (4.21, 7.29)
Wolf 2020 (60)	No	Capmatinib (84, with GCN ≥10^a^)	Phase II	• 29% (1–2 previous lines of therapy) • 40% (no previous lines of therapy)	• 4.1 (2.9, 4.8) (1–2 previous lines of therapy) • 4.2 (1.4, 6.9) (no previous lines of therapy)
Dagogo-Jack 2021 (54)	No	Capmatinib (5)	Phase II	0%	Not reported
Yang 2021 (76)	Yes	Savolitinib + gefitinib (51)	Phase Ib	31%	4.0 (2.8, 5.5)
Sequist 2020 (57)	Yes	Savolitinib + osimertinib (180)	Phase Ib	48% (Cohort B)^b^ 64% (Cohort D)^b^	7.6 (5.5, 9.2) (Cohort B)^b^ 9.1 (5.4, 12.9) (Cohort D)^b^
Yu 2021 (77)	Yes	Savolitinib + osimertinib (17)	Phase II	41%	Not reported
Camidge 2021 (30)	No	Crizotinib (38)	Phase I	28.9%	5.1 (1.9, 7.0)
Landi 2019 (55)	No	Crizotinib (16)	Phase II	31.3%	5.0 (2.7, 7.3)
Angevin 2017 (58)	No	SAR125844 (22)	Phase I	18.2%	Not reported

a Cohorts with GCN <10 were closed for futility.

b Patients included in this trial had locally advanced or metastatic *MET*-amplified, *EGFR* mutation-positive NSCLC, who had progressed on EGFR-TKI. Cohort B comprised three prespecified sub-cohorts (sub-cohort B1 included patients who had received previous treatment with a third-generation EGFR-TKI, and sub-cohorts B2 and B3 included patients who had not received previous treatment with a third-generation EGFR-TKI; patients in B2 were *EGFR* T790M-negative at enrollment whereas patients in B3 were T790M-positive at enrollment. Cohort D included patients who had received previous treatment with first-generation or second-generation EGFR-TKIs (and no third-generation EGFR-TKIs), and who were *EGFR* T790M-negative at study enrollment.

CI, confidence interval; CR, complete response; EGFR, epidermal growth factor receptor; GCN, gene copy number; *MET*amp, mesenchymal-epithelial transition factor amplification; NSCLC, non-small cell lung cancer; ORR, objective response rate; PFS, progression-free survival; PR, partial response; TKI, tyrosine kinase inhibitor.

##### 3.5.1.1 MET-TKI combination therapy for the treatment of secondary *MET*amp NSCLC

###### 3.5.1.1.1 Capmatinib

Results from a single arm Phase Ib/II trial evaluating capmatinib plus gefitinib after failure of EGFR inhibitor therapy in patients with *EGFR*-mutated, *MET*amp NSCLC (n=36) demonstrated that patients treated with capmatinib plus gefitinib had a median (95% CI) progression-free survival of 5.49 (4.21, 7.29) months and an objective response rate of 47% (56).

###### 3.5.1.1.2 Savolitinib

Results from a Phase Ib trial evaluating savolitinib plus gefitinib after progression on an EGFR-TKI in patients with *EGFR*-mutated, *MET*amp advanced NSCLC (n=51) demonstrated that patients treated with savolitinib plus gefitinib had a median (95% CI) progression-free survival of 4.0 (2.8, 5.5) months and an objective response rate of 31% (76).

Another Phase Ib trial (TATTON) evaluated savolitinib plus gefitinib after progression on an EGFR-TKI in patients with *EGFR*-mutated, *MET*amp advanced NSCLC in two expansion cohorts (57). The TATTON trial was divided into four parts, A to D, and parts B and D were the two global expansion cohorts evaluating savolitinib plus gefitinib. Part B consisted of three cohorts of patients: those who had been previously treated with a third-generation EGFR-TKI and those who had not been previously treated with a third-generation EGFR-TKI who were either T790M negative or T790M positive; part D enrolled patients who had not previously received a third-generation EGFR-TKI and were T790M negative. Results from this study demonstrated that the median (95% CI) progression-free survival in cohort B (n=138) was 7.6 (5.5, 9.2) months and for cohort D (n=42) was 9.1 (5.4, 12.9) months. The objective response rate was 48% in cohort B and 64% in cohort D.

Savolitinib in combination with osimertinib is being evaluated in several Phase II and III ongoing trials (94, 100–103). Results from the Phase II ORCHARD study evaluating savolitinib plus osimertinib after progression on first-line osimertinib monotherapy among patients with locally advanced/metastatic *EGFR*-mutant NSCLC demonstrated that patients treated with savolitinib plus osimertinib (n=17) had an overall response rate of 41% after a follow-up of 13 weeks (77). Results for other Phase II and III studies evaluating savolitinib plus osimertinib were not identified at the time of this literature review.

###### 3.5.1.1.3 Tepotinib

A total of 19 patients with *EGFR*-mutated, *MET*amp advanced NSCLC who progressed on an EGFR-TKI were enrolled in a Phase Ib/II trial (INSIGHT) (61, 78). In this study evaluating tepotinib plus gefitinib (n=12) and chemotherapy (n=7), median (90% CI) progression-free survival was 16.6 (8.3, 22.1) and 4.2 (1.4, 7.0) months (hazard ratio: 0.13; 90% CI: 0.04, 0.43), and median (90% CI) overall survival was 37.3 (21.1, 52.1) and 13.1 (3.3, 22.6) months (hazard ratio: 0.10; 90% CI: 0.02, 0.36). The objective response rate was 67% in patients receiving tepotinib plus gefitinib and 43% in patients receiving chemotherapy (78).

Tepotinib in combination with osimertinib is also being evaluated among patients with *EGFR-*mutated NSCLC following progression on osimertinib in the Phase II INSIGHT-2 trial (99). Primary analysis results for INSIGHT-2 have been presented at the World Conference on Lung Cancer Congress in September 2023 but were not yet published at the time of this literature review (119).

##### 3.5.1.2 MET-TKI monotherapy

###### 3.5.1.2.1 Bozitinib

Preliminary results from a Phase I, open-label, multicenter study evaluating bozitinib in locally advanced or metastatic NSCLC with MET dysregulation demonstrated that bozitinib had a manageable safety profile at the recommended Phase II dose of 200 mg twice daily (120). Only eight patients had *MET*amp NSCLC in this study, and it was not specified if patients had primary or secondary *MET*amp.

###### 3.5.1.2.2 Capmatinib

Two Phase II trials evaluated the use of capmatinib monotherapy in patients with *MET*amp NSCLC (54, 60). In the first study, a Phase II trial evaluating capmatinib in *MET* exon 14-mutated or *MET*amp NSCLC, patients did not have an *EGFR* mutation or *ALK* fusion (60). Patients with *MET*amp NSCLC and a gene copy number ≥10 had a median (95% CI) progression-free survival of 4.1 (2.9, 4.8) months for patients who had received 1–2 previous lines of therapy (n=69) and 4.2 (1.4, 6.9) months for patients with no previous lines of therapy (n=15) (60). The objective response rate was 29% for patients with 1–2 previous lines of therapy and 40% for patients with no previous lines of therapy. Additional cohorts for patients with a gene copy number less than 10 (gene copy number 6–9, 4–5, or < 4) were initially planned and ultimately closed for futility at an interim analysis (60).

In the second study, a Phase II trial evaluating capmatinib in patients with advanced NSCLC harboring *MET*amp or *MET* exon 14 skipping alterations, patients must have received treatment with a prior MET-TKI, and there was no restriction on the number of prior treatment regimens (54). Only five patients in this trial had *MET*amp, and none of these five patients achieved an objective response (54). The authors noted that a limitation of this study was the limited number of patients enrolled with *MET*amp NSCLC.

###### 3.5.1.2.3 Crizotinib

In a Phase I study enrolling patients with advanced NSCLC who have not received previous hepatocyte growth factor- or MET-targeted therapy, *MET*amp was defined as a *MET/CEP7* ratio >1.8; low levels of *MET*amp were defined as ≥1.8 to ≤2.2; medium levels as >2.2 to < 4.0; and high levels as ≥4.0 (the initial cut-off for the high versus medium threshold was changed from a ≥5 to ≥4 *MET/CEP7* ratio based on a preliminary response analysis) (30). Results from this study demonstrated that patients with advanced *MET*amp NSCLC had a median (95% CI) progression-free survival of 5.1 (1.9, 7.0) months for all patients (N=38), 6.7 (3.4, 9.2) months for patients with high *MET*amp (n=21), 1.9 (1.3, 5.6) months for patients with medium *MET*amp (n=14), and 1.8 (0.8, 14.0) months for patients with low *MET*amp (n=3). The objective response rate (95% CI) was 28.9% (15.4, 45.9) for all patients, 38.1% (18.1, 61.6) in the high *MET*amp group, 14.3% (1.8, 42.8) in the medium *MET*amp group, and 33.3% (0.8, 90.6) in the low *MET*amp group. Median overall survival (95% CI) with crizotinib was 11.0 (7.1, 15.9) months for all patients, 11.4 (7.2, 19.3) months for patients with high *MET*amp, 9.2 (2.1, 18.1) months for patients with medium *MET*amp, and 5.6 (1.1, not estimable) months for patients with low *MET*amp.

In a Phase II trial, crizotinib monotherapy was evaluated in patients with pretreated NSCLC with *MET* dysregulation or evidence of *ROS1* rearrangements; patients with *EGFR* or *KRAS* mutations were excluded (55). Patients with *MET*amp NSCLC (n=16) had a median (95% CI) progression-free survival of 5.0 (2.7, 7.3) months, a median (95% CI) overall survival of 5.4 (3.4, 7.4) months, and an objective response rate of 31.3% (95% CI: 5.2, 71.4) (55).

###### 3.5.1.2.4 SAR125844

Results from a first-in-human Phase I trial evaluating SAR125844 in advanced solid tumors and *MET* dysregulation found that patients with advanced *MET*amp NSCLC (n=22) demonstrated a partial response rate of 18.2%; no patients achieved a complete response (58). Patients in this trial were allowed, but not required, to have *EGFR* mutations or prior EGFR inhibitor therapy.

###### 3.5.1.2.5 Tepotinib

Results from a Phase II trial evaluating tepotinib in *EGFR* and *ALK*-wild type NSCLC with high-level *MET*amp (VISION) demonstrated that tepotinib (n=24) showed clinical activity, especially in the first-line setting at the data cut-off (August 20, 2021) (62, 121). Among all patients, the objective response rate was 41.7% (95% CI: 22.1, 63.4) and the median duration of response was 14.3 months (95% CI: 2.8, not estimable). Among patients treated in the first-line setting (n=7), the objective response rate was 71.4% (95% CI: 29.0, 96.3) and the median duration of response was 14.3 months (95% CI: 2.8, not estimable). Results at the August 20, 2021 data cut-off, identified through this literature search, were initially presented as conference proceedings in 2022 (62) and subsequently published in November 2023 (121).

#### 3.5.2 Anti-MET antibodies

##### 3.5.2.1 Amivantamab

Amivantamab has a unique structure in that it is a bispecific antibody that blocks both epidermal growth factor and MET receptors (80). It is being investigated in multiple studies, including in a Phase I trial enrolling patients with metastatic or unresectable NSCLC that has progressed after prior standard of care therapy (CHRYSALIS-1). The objective of this study is to evaluate the safety, pharmacokinetics, and preliminary efficacy of amivantamab (either alone or in combination with lazertinib), and to determine the recommended Phase II doses for expansion of amivantamab monotherapy and combination therapy. One of the cohorts includes patients with primary *EGFR*-mutated disease and documented *MET*amp or *MET* mutation after progression on any EGFR-TKI. Published results for patients with *MET*amp NSCLC from this trial were not identified at the time of the literature review.

##### 3.5.2.2 Sym015

Sym015 is a mixture of two humanized antibodies targeting MET (59). Interim safety and efficacy results from a Phase I trial were reported for eight patients with advanced *MET*amp NSCLC (including seven patients who were MET-TKI-naïve, and one patient previously treated with a MET-TKI). The objective response rate was 25%.

#### 3.5.3 Anti-MET antibody–drug conjugates

##### 3.5.3.1 Telisotuzumab vedotin

Telisotuzumab vedotin is a first-in-class antibody–drug conjugate consisting of a humanized MET-targeting antibody, ABT-700, coupled to a cytotoxic microtubule inhibitor, monomethyl auristatin E, through a valine–citrulline linker (81). It is currently being evaluated as monotherapy and in combination with osimertinib, erlotinib, and nivolumab in participants with advanced solid tumors, including *MET*amp NSCLC (98).

## 4 Discussion

Results from this review indicate that primary *MET*amp occurs in approximately 5% of NSCLC cases. Secondary *MET*amp, which is an established mechanism of resistance for patients with *EGFR* mutation-positive NSCLC (3), occurs in approximately 15% of advanced NSCLC cases previously treated with an EGFR inhibitor in the US. *MET*amp has also been identified as a mechanism of resistance in patients with NSCLC and other actionable genomic alterations, including *ALK* fusion, *RET* fusion, *ROS1* fusion, and *KRAS* G12C mutation (38, 40, 122–127). An overview of the MET pathway and mechanisms of *MET*amp-mediated resistance in NSCLC is not included in the scope of this literature review and has been previously described in several reviews (3, 128–130).

The higher rates of secondary *MET*amp among patients with *EGFR-*mutant NSCLC compared with primary *MET*amp in NSCLC indicate a need for testing at different time points of the treatment journey, including at diagnosis and upon progression on an EGFR-TKI. In real-world settings, FISH and NGS are both commonly used to test for *MET*amp. Variability exists in how *MET*amp is defined across clinical trials and real-world evidence studies. Further studies are needed to evaluate the use and reliability of different testing strategies (NGS vs FISH; and tumor vs liquid testing) for identification of patients with *MET*amp, with potential advantage to improved standardization of definitions of *MET*amp.

No studies evaluating the economic and humanistic burdens of *MET*amp NSCLC were identified in this literature review, suggesting further studies evaluating these burdens are warranted. Furthermore, there is a lack of US-based evidence on treatments used in real-world settings following diagnosis of *MET*amp NSCLC. Any interpretation of real-world evidence is limited as sample sizes were small in the few studies identified. Studies evaluating real-world practice patterns and outcomes among patients with *MET*amp NSCLC in the US and other countries are needed.

As of March 2023, there were no US Food and Drug Administration approved therapies for *MET*amp NSCLC. The NCCN Guidelines recommend capmatinib, crizotinib, and tepotinib as monotherapy for patients with metastatic NSCLC and high-level *MET*amp (4). The NCCN Guidelines also note that the best management of any patient with cancer is in a clinical trial, and participation in clinical trials is especially encouraged (4). The European Society for Medical Oncology guidelines similarly note that while *MET*amp is a promising therapeutic target, targeting *MET*amp is not currently routinely recommended and recruitment into trials is encouraged (6).

Historically, trials aimed at targeting MET overexpression (e.g., onartuzumab) in NSCLC have failed (6). Onartuzumab (METmab), for example, is a MET-receptor monoclonal antibody that was evaluated in combination with erlotinib for the treatment of advanced NSCLC with MET diagnostic-positive status tested by immunohistochemistry; however, the Phase III trial evaluating onartuzumab combination therapy was stopped prematurely due to lack of clinically meaningful efficacy (131).

Instead of MET overexpression, the focus has shifted to targeting genomic variants, such as *MET* exon 14 skipping and *MET*amp (6). Several agents alone or in combination with EGFR-TKIs were identified as under investigation for the treatment of *MET*amp NSCLC. Information on ClinicalTrials.gov is evolving and additional trials evaluating therapies for *MET*amp NSCLC have been indexed since the time of our literature search in June 2022, such as the Phase I trial evaluating the antibody-drug conjugate MYTX-011 (132) and a Phase I/II trial evaluating amivantamab and capmatinib combination therapy (133).

Concomitant inhibition of both *EGFR* and *MET* is thought to overcome resistance to EGFR inhibitors due to *MET*amp (9, 134), and combination therapy with EGFR inhibitors and several type I MET-TKIs (tepotinib, savolitinib, and capmatinib) have shown promising results among patients with *EGFR*-mutated, *MET*amp NSCLC who progressed on a prior EGFR-TKI (56, 76, 78, 119, 135). Final analysis of the Phase Ib/II INSIGHT trial demonstrated an objective response rate of 66.7% in patients treated with tepotinib plus gefitinib (78). Primary analysis results from the Phase II INSIGHT-2 trial (data cut-off: March 28, 2023; results were published in 2023 after our literature search was performed) demonstrated that treatment with tepotinib plus osimertinib demonstrated an objective response rate of 50.0% and a median progression-free survival of 5.6 months among patients with *EGFR-*mutant NSCLC and *MET*amp who progressed on first-line osimertinib (119). Preliminary results from the SAVANNAH Phase II trial published in 2022 showed that savolitinib plus osimertinib demonstrated an objective response rate of 49% in patients with *EGFR-*mutated NSCLC with high levels of *MET*amp, defined as FISH 10+, whose disease progressed on treatment with osimertinib (135). Results from a Phase Ib/II study showed that patients treated with capmatinib plus gefitinib had an objective response rate of 47% (56).

### 4.1 Limitations

The scope of the literature review search strategies included terms for NSCLC, *MET*amp, and outcomes of interest. Due to inclusion of *MET*amp terms in the search strategies, references describing rates of secondary *MET*amp as a secondary or exploratory outcome among patients with *EGFR*-mutant NSCLC, references describing broad molecular testing patterns for all actionable driver mutations in NSCLC, or references describing treatments under investigation for patients with *MET* alterations generally (and not *MET*amp specifically) may not have been captured. The included data were current as of our search date (June 14, 2022); data published or included in a database after that date were not captured. Treatments under investigation for *MET*amp NSCLC are evolving rapidly, and ClinicalTrials.gov should be checked regularly for an up-to-date list of ongoing trials. Information available on ClinicalTrials.gov, however, is limited and not all actionable genomic alterations being evaluated in a clinical trial and included in a study protocol may be listed on ClinicalTrials.gov.

## 5 Conclusion

Results from this literature review demonstrate that primary *MET*amp occurs in approximately 5% of NSCLC cases and secondary *MET*amp occurs in approximately 15% of advanced NSCLC cases previously treated with an EGFR inhibitor in the US. NGS and FISH were commonly used to identify *MET*amp in real-world studies; across clinical trials, variability existed in how *MET*amp was defined. Future studies evaluating the economic and humanistic burden, as well as the real-world evidence on treatment for advanced *MET*amp NSCLC are needed.

Several promising agents, including MET-TKIs in combination with EGFR-TKIs, are currently under investigation for secondary *MET*amp NSCLC.

## Data availability statement

The original contributions presented in the study are included in the article/**Supplementary Material.** Further inquiries can be directed to the corresponding author.

## Author contributions

All authors contributed to the conception or design of the work; the acquisition, analysis, or interpretation of data for the work; drafting the work or revising it critically for important intellectual content; providing final approval of the version to be published; and agree to be accountable for all aspects of the work in ensuring that questions related to the accuracy or integrity of any part of the work are appropriately investigated and resolved. All authors contributed to the article and approved the submitted version.
