# Supplementary material for: Non-small cell lung cancer with MET amplification: review of epidemiology, associated disease characteristics, testing procedures, burden, and treatments
Source: Front Oncol. 2024 Jan 11;13:1241402. doi: 10.3389/fonc.2023.1241402 (PMC10808753; doi:10.3389/fonc.2023.1241402)
Supplement: Supplementary file 1 [file Table_1.docx]

Supplementary Material

Non-small cell lung cancer with *MET* amplification: Review of epidemiology, associated disease characteristics, testing procedures, burden, and treatments

**Mo Yang*, Erin Mandal, Frank X. Liu, Richard M. O’Hara, Jr., Beth Lesher, Rachel E. Sanborn**

*** Correspondence:** Mo Yang: [mo.yang@emdserono.com](mailto:mo.yang@emdserono.com)

# Supplementary Data

Supplemental Table 1. Search conducted via ProQuest (for MEDLINE and Embase) on June 14, 2022

| Set # | Searched for | Results |
| --- | --- | --- |
| S1 – NSCLC | (EMB.EXACT.EXPLODE("non small cell lung cancer") OR MESH.EXACT.EXPLODE("Carcinoma, Non-Small-Cell Lung") OR TI,AB("non small cell lung cancer") OR TI,AB("NSCLC")) | 275,092^a^ |
| S2 – *MET* terms + amplification | ((EMB.EXACT.EXPLODE("epithelial mesenchymal transition") OR MESH.EXACT.EXPLODE("Epithelial-Mesenchymal Transition") OR TI,AB(“mesenchymal epithelial transition”) OR TI,AB(MET)) AND (EMB.EXACT.EXPLODE("gene amplification") OR MESH.EXACT.EXPLODE("Gene Amplification") OR TI,AB(amplification OR amplified))) OR TI,AB(METamp) | 10,033^a^ |
| S3 – combined | S1 AND S2 | 2,279^b^ |
| S4 – limits (language) | S3 AND LA(English) | 2,234^b^ |
| S5 – limits (publication type) | S4 NOT (EMB.EXACT(editorial OR “case report” OR letter OR note) OR DTYPE("Editorial" OR "Comment" OR "Letter" OR "Case Reports" OR "News" OR "Newspaper Article") OR TI,AB(“case study” or “case studies” OR “case report*” OR “case series”)) | 2,009^b^ |
| S6 – limits (publication type) | S5 NOT (rtype.exact("Conference Abstract")) | 899^b^ |
| S7 – limits (publication type) | S5 AND (rtype.exact("Conference Abstract")) | 1,114^b^ |
| S8 – limits (date) | S6 AND pd(>20141231) | 667^b^ |
| S9 – limits (date) | S7 AND pd(>20191231) | 182^b^ |
| S10 – total | S8 OR S9 | 849^b^ |
| ^a^ Duplicates are removed from the search but included in the result count.  ^b^ Duplicates are removed from the search and from the result count. | | |

Supplemental Table 2. Search conducted via ClinicalTrials.gov on June 14, 2022

| Searched for | Results |
| --- | --- |
| ((NSCLC OR “non small cell lung cancer”) AND (((“mesenchymal epithelial transition” OR MET) AND (amplification OR amplified)) OR (METamp))) | 62 trials |

Supplemental Table 3. Search conducted via Cochrane Controlled Register of Trials (CENTRAL) on June 14, 2022

| Searched for | Results |
| --- | --- |
| Title Abstract Keyword search: ((NSCLC OR “non small cell lung cancer”) AND (((“mesenchymal epithelial transition” OR MET) AND (amplification OR amplified)) OR (METamp))) | 72 trials |

Supplemental Table 4. Summary of statistical associations between presence of *MET*amp in NSCLC and demographic and clinical characteristics reported in 16 identified trials

| Reference | Country | NSCLC patient group | Summary of associations between demographic or clinical characteristics and presence of *MET*amp (as reported by study authors) |
| --- | --- | --- | --- |
| **Studies enrolling patients with NSCLC; patients were not required to have *EGFR* mutations or prior progression on an EGFR-TKI** | | | |
| Ando 2019 (1) | Japan | - *MET*amp (n=9) - No *MET*amp (n=79) | - Patients with *MET*amp vs no *MET*amp had no significant differences in median age (p=0.166), sex (p=0.270), or smoking status (p=0.220) |
| Albitar 2018 (2) | United States | - *MET*amp (n=16) - No *MET*amp (n=373) | - Patients with *MET*amp vs no *MET*amp had a significantly higher percentage of PD-L1 expression expressed as a continuous variable (p=0.004), as well as when cut-off points of 5% (p=0.01), 20% (p=0.0006), and 50% (p=0.01) were used |
| Burns 2021 (3) | Not reported | - Lung adenocarcinoma brain metastases samples (n=125) - Primary lung adenocarcinoma samples (n=447) | - *MET*amp was observed at a significantly higher rate among brain metastases samples vs primary samples (19% vs 3%; p<0.00001) |
| Chan 2018 (4) | Macao | - *MET*amp (n=3) - No *MET*amp (n=119) | - Patients with *MET*amp vs no *MET*amp had no significant differences in age (p=0.118), sex (p=0.429), NSCLC subtype (adenocarcinoma vs non-adenocarcinoma; p=0.642), smoking status (p=0.636), or presence of liver metastases (p=0.204) |
| Chen 2015 (5) | China | *MET*amp tested with tumor tissue   - *MET*amp (n=23) - No *MET*amp (n=234)   *MET*amp tested with peripheral blood samples   - *MET*amp (n=31) - No *MET*amp (n=287) | Among patients tested for *MET*amp with tumor tissue:   - Patients with *MET*amp vs no *MET*amp had no significant differences in age (p=0.401), sex (p=0.321), or NSCLC subtype (adenocarcinoma vs non-adenocarcinoma; p=0.160) - Rates of *MET*amp were significantly higher among patients with a positive smoking history vs no *MET*amp (p=0.029)   Among patients tested for *MET*amp with peripheral blood samples:   - Patients with *MET*amp vs no *MET*amp had no significant differences in age (p=0.664), or NSCLC subtype (adenocarcinoma vs non-adenocarcinoma; p=0.091) - Rates of *MET*amp were significantly higher among male patients (p=0.026) and patients with a positive smoking history (p=0.005) |
| Domènech 2021 (6) | Spain | - *MET*amp (n=14) - No *MET*amp (n=33) | - A higher proportion of positive PD-L1 expression was found among patients with *MET*amp (93%) vs non-*MET*amp (42%; p<0.001) |
| Fang 2018 (7) | United States | - *MET*amp (n=29) - No *MET*amp (n=338) - *MET* copy number gain (n=17) | - Patients with *MET*amp vs no *MET*amp or *MET* copy number gain had no significant differences in median age at diagnosis (p=0.338), sex (p=0.549), or NSCLC subtype (adenocarcinoma vs squamous cell carcinoma or other; p=0.364) - Patients with *MET*amp vs no *MET*amp had a significantly shorter time to distant metastasis (11.6 vs 43.8 months; p=0.004) - *MET*amp was highly associated with distant metastases (hazard ratio: 4.86; 95% CI: 1.85, 12.75; p=0.001) |
| Huang 2022 (8) | United States | - NSCLC brain metastases samples (n=3,035) - Primary NSCLC samples (n=7,277) | - *MET*amp was observed at a significantly higher rate among NSCLC brain metastases samples vs primary NSCLC samples (4.4% vs 2.3%; p<0.05) |
| Park 2015 (9) | South Korea | - *MET*amp (n=26) - No *MET*amp (n=290) | - Patients with *MET*amp vs no *MET*amp had no significant differences in age (p=0.050), sex (p=0.758), or smoking status (p=0.844) |
| Peng 2022 (10) | China | - *MET*amp/*EGFR*-mutation positive (n=18) - No *MET*amp/*EGFR*-mutation positive (n=22) | - Patients with *MET*amp vs no *MET*amp had no significant differences in age (p=0.106), sex (p=0.526), smoking status (p=1.000), or presence of brain metastases (p=0.498) |
| Shi 2021 (11) | Multi-country | - Lung adenocarcinoma metastases samples (n=2,915) - Primary lung adenocarcinoma samples (n=4,448) | - *MET*amp was observed at a significantly higher rate among metastases samples vs primary samples (11.6% vs 5.7%; p<0.001) |
| Song 2017(12) | China | - *MET*amp (n=8) - No *MET*amp (n=783) | - Patients with *MET*amp vs no *MET*amp had no significant differences in age (p=0.95), sex (p=0.80), NSCLC subtype (adenocarcinoma vs non-adenocarcinoma; p=0.85), or smoking status (p=0.96) |
| Xu 2017(13) | China | - *MET*amp (n=33) - No *MET*amp (n=335) | - Patients with *MET*amp vs no *MET*amp had no significant differences in age (p=0.130), sex (p=0.177), NSCLC subtype (adenocarcinoma vs squamous cell carcinoma; p=0.064), or smoking status (p=0.087) |
| Yoshimura 2021(14) | Japan | - *MET*amp (n=13) - No *MET*amp (n=162) | - Patients with *MET*amp vs no *MET*amp had no significant differences in age (p=0.131), sex (p=1.000), NSCLC subtype (adenocarcinoma vs squamous cell carcinoma; p=0.410), or smoking status (p=1.000) |
| **Studies enrolling patients with NSCLC who had advanced *EGFR-*mutant NSCLC and were tested for *MET*amp after EGFR-TKI failure** | | | |
| Ahn 2021(15) | South Korea | - *MET*amp (n=30) - No *MET*amp (n=156) | - Patients with *MET*amp vs no *MET*amp had no significant differences in median age (p=0.362), sex (p=0.482), or rates of progression to liver metastases (p=0.185) - Patients with *MET*amp vs no *MET*amp were significantly more likely to be a current or ex-smoker (53.3% vs 28.8%; p=0.013) - Patients with *MET*amp vs no *MET*amp were significantly less likely to have progression with brain metastases (p<0.001) |
| Baldacci 2017(16) | France | - *MET*amp (n=19) - No *MET*amp and MET overexpression (n=17) | - Patients with *MET*amp vs no *MET*amp/MET overexpression had no significant differences in median age (p=0.73), sex (p=0.35), or smoking status (p=0.45) |

EGFR, epidermal growth factor receptor; *MET*amp, mesenchymal-epithelial transition factor amplification; NSCLC, non-small cell lung cancer; PD-L1, programmed death-ligand 1; TKI, tyrosine kinase inhibitor.

Supplemental Table 5. Real-world evidence studies evaluating treatment patterns and outcomes in patients with advanced *MET*amp NSCLC

| Reference | Country | Treatment |
| --- | --- | --- |
| **NSCLC and secondary *MET*amp** | | |
| Baldacci 2017 (16) | France | - Crizotinib (n=12) - Osimertinib (n=5) |
| Fuchs 2021 (17) | Israel | - Osimertinib + MET-targeted therapy (n=2) |
| Li 2020^a^ (18) | China | - Crizotinib (n=9) |
| Liu 2021 (19) | China | - Crizotinib + EGFR-TKI (n=38) - Crizotinib monotherapy (n=10) - Chemotherapy (n=22) |
| Wang 2020 (20) | China | - Gefitinib + crizotinib^b^ (n=3) - Erlotinib + crizotinib^b^ (n=1) - Osimertinib + crizotinib^c^ (n=5) - Icotinib + crizotinib^c^ (n=1) - Gefitinib + crizotinib^c^ (n=1) |
| Wang 2019 (21) | China | - Crizotinib + EGFR-TKI (n=6) - Crizotinib monotherapy (n=8) - Chemotherapy (n=4) |
| **NSCLC and primary *MET*amp** | | |
| Li 2020^a^ (18) | China | - Crizotinib (n=10) |
| Liu 202^d^ (22) | China | - MET-TKIs (crizotinib, n=25 or bozitinib, n=4) |
| Mi 2022^e^ (23) | China | - EGFR-TKI monotherapy (n=19) - EGFR-TKI + crizotinib (n=9) |
| Song 2019^d^ (24) | China | - Crizotinib (n=15) |
| Tsui 2022^f^ (25) | United States | - Crizotinib (n=30) or capmatinib (n=9) |
| **NSCLC with either primary or secondary *MET*amp (not specified or results presented together)** | | |
| Bittoni 2021 (26) | Multicountry^g^ | - 1L (n=25) and 2L (n=18) therapy |
| Kron 2021^h^ (27) | Germany | - ICI monotherapy (n=98) - Chemotherapy (n=124) |
| Shalata 2022 (28) | Israel | - Crizotinib (n=9) |
| Suryavanshi 2017 (29) | India | - Crizotinib (n=4) |
| Tacar 2022 (30) | Turkey | - Crizotinib (n=8) |
| Yoshimura 2021^i^ (14) | Japan | - Nivolumab (n=13) |

^a^ Study includes data for both primary and secondary *MET*amp presented separately.

^b^ Given after progression on 1L gefitinib.

^c^ Given after relapse on 2L gefitinib.

^d^ Patients previously treated with EGFR-TKIs before MET detection were excluded.

^e^ Patients with concurrent *EGFR*-mutant and primary *MET*amp were included.

^f^ *MET*amp could co-occur with other biomarkers, but it was not an inclusion criteria.

^g^ Israel, The Netherlands, Taiwan, and the United States.

^h^ Concurrent mutations include: *TP53*, *KRAS*, *BRAF*, *KEAP1*, *NFE2L2*, *PIK3CA*, and *NRAS*.

^I^ Exploratory post hoc analysis to evaluate the association of *MET*amp with efficacy of patients with advanced or recurring NSCLC treated with nivolumab monotherapy.

*MET*amp, mesenchymal-epithelial transition factor amplification; NSCLC, non-small cell lung cancer; MET, mesenchymal-epithelial transition factor; *EGFR*, epidermal growth factor receptor; TKI, tyrosine kinase inhibitor; 1L, first-line; 2L, second-line; ICI, immune checkpoint inhibitor*; TP53*, tumor protein 53; *KRAS,* Kirsten rate sarcoma viral oncogene homolog; *BRAF,* B-Raf proto-oncogene serine/threonine kinase; *KEAP1,* Kelch-like ECH-associated protein 1; *NFE2L2,* nuclear factor, erythroid 2-like 2; *PIK3CA*, phosphatidylinositol-4,5-bisphosphate 3-kinase catalytic subunit alpha; *NRAS,* neuroblastoma ras viral oncogene homolog.

# References

1. Ando R, Fujino M, Kominami-Kiriyama A, Ito A, Koide T, Ito M. Mesenchymal–epithelial transition gene amplification and protein overexpression in stage IV pulmonary adenocarcinoma. Jpn J Clin Oncol (2019) 49(8):755-61. doi: 10.1093/jjco/hyz060

2. Albitar M, Sudarsanam S, Ma W, Jiang S, Chen W, Funari V, et al. Correlation of MET gene amplification and TP53 mutation with PD-L1 expression in non-small cell lung cancer. Oncotarget (2018) 9(17):13682-93. doi: 10.18632/oncotarget.24455

3. Burns TF, Dacic S, Velez MA, Somasundaram A, Bhattacharya S, Chakka A, et al. MET alterations are enriched in lung adenocarcinoma brain metastases and define a distinct molecular and transcriptomic subtype. Cancer Research (2021) 81(13_Supplement):2218. doi: 10.1158/1538-7445.AM2021-2218

4. Chan KI, Vong HT, Sin LF, Yip YC, Zhong XY, Wen JM. Relationship between driver gene mutations, their relative protein expressions and survival in non-small cell lung carcinoma in Macao. Clin Respir J (2018) 12(4):1416-23. doi: 10.1111/crj.12670

5. Chen D, Xu C, Wu J, Zhang Y, Fang M. A comparison of consistency of detecting c-MET gene amplification in peripheral blood and tumor tissue of nonsmall cell lung cancer patients. J Cancer Res Ther (2015) 11(5):C63-C7. doi: 10.4103/0973-1482.163843

6. Domènech M, Muñoz Marmol AM, Mate JL, Estival A, Moran T, Cucurull M, et al. Correlation between PD-L1 expression and MET gene amplification in patients with advanced non-small cell lung cancer and no other actionable oncogenic driver. Oncotarget (2021) 12(18):1802-10. doi: 10.18632/oncotarget.28045

7. Fang L, Chen H, Tang Z, Kalhor N, Liu C-H, Yao H, et al. MET amplification assessed using optimized FISH reporting criteria predicts early distant metastasis in patients with nonsmall cell lung cancer. Oncotarget (2018) 9(16):12959-70. doi: 10.18632/oncotarget.24430

8. Huang RSP, Harries L, Decker B, Hiemenz MC, Murugesan K, Creeden J, et al. Clinicopathologic and genomic landscape of non-small cell lung cancer brain metastases. Oncologist (2022) 27(10):839-48. doi: 10.1093/oncolo/oyac094

9. Park S, Koh J, Kim D-W, Kim M, Keam B, Kim TM, et al. MET amplification, protein expression, and mutations in pulmonary adenocarcinoma. Lung Cancer (2015) 90(3):381-7. doi: 10.1016/j.lungcan.2015.10.022

10. Peng K-C, Su J-W, Xie Z, Wang H-M, Fang M-M, Li W-F, et al. Clinical outcomes of EGFR+/METamp+ vs. EGFR+/METamp- untreated patients with advanced non-small cell lung cancer. Thorac Cancer (2022) 13(11):1619-30. doi: 10.1111/1759-7714.14429

11. Shi H, Seegobin K, Heng F, Zhou K, Zhao Y, Manochakian R, et al. Genomic characterization of primary versus metastatic lung adenocarcinoma. J Thorac Oncol (2021) 16(10):S973. doi: 10.1016/j.jtho.2021.08.258

12. Song Z, Wang X, Zheng Y, Su H, Zhang Y. MET gene amplification and overexpression in Chinese non–small-cell lung cancer patients without EGFR mutations. Clin Lung Cancer (2017) 18(2):213-9.e2. doi: 10.1016/j.cllc.2016.09.011

13. Xu C-W, Wang W-X, Wu M-J, Zhu Y-C, Zhuang W, Lin G, et al. Comparison of the c-MET gene amplification between primary tumor and metastatic lymph nodes in non-small cell lung cancer. Thorac Cancer (2017) 8(5):417-22. doi: 10.1111/1759-7714.12455

14. Yoshimura K, Inoue Y, Inui N, Karayama M, Yasui H, Hozumi H, et al. MET amplification and efficacy of nivolumab in patients with NSCLC. JTO Clin Res Rep (2021) 2(11):100239. doi: 10.1016/j.jtocrr.2021.100239

15. Ahn B-C, Lee JH, Kim MH, Pyo K-H, Lee C-K, Lim SM, et al. Distinct characteristics and clinical outcomes to predict the emergence of met amplification in patients with non-small cell lung cancer who developed resistance after treatment with epidermal growth factor receptor tyrosine kinase inhibitors. Cancers (2021) 13(12):3096. doi: 10.3390/cancers13123096

16. Baldacci S, Mazieres J, Tomasini P, Girard N, Guisier F, Audigier-Valette C, et al. Outcome of EGFR-mutated NSCLC patients with MET-driven resistance to EGFR tyrosine kinase inhibitors. Oncotarget (2017) 8(62):105103-14. doi: 10.18632/oncotarget.21707

17. Fuchs V, Roisman L, Kian W, Daniel L, Dudnik J, Nechushtan H, et al. The impact of osimertinib’ line on clonal evolution in EGFRm NSCLC through NGS-based liquid biopsy and overcoming strategies for resistance. Lung Cancer (2021) 153:126-33. doi: 10.1016/j.lungcan.2020.12.039

18. Li J, Wang Y, Zhang B, Xu J, Cao S, Zhong H. Characteristics and response to crizotinib in lung cancer patients with MET amplification detected by next-generation sequencing. Lung Cancer (2020) 149:17-22. doi: 10.1016/j.lungcan.2020.08.021

19. Liu L, Qu J, Heng J, Zhou C, Xiong Y, Yang H, et al. A large real-world study on the effectiveness of the combined inhibition of EGFR and MET in EGFR-mutant non-small-cell lung cancer after development of EGFR-TKI resistance. Front Oncol (2021) 11:722039. doi: 10.3389/fonc.2021.722039

20. Wang Y, Tian P, Xia L, Li L, Han R, Zhu M, et al. The clinical efficacy of combinatorial therapy of EGFR-TKI and crizotinib in overcoming MET amplification-mediated resistance from prior EGFR-TKI therapy. Lung Cancer (2020) 146:165-73. doi: 10.1016/j.lungcan.2020.06.003

21. Wang W, Wang H, Lu P, Yu Z, Xu C, Zhuang W, et al. Crizotinib with or without an EGFR-TKI in treating EGFR-mutant NSCLC patients with acquired MET amplification after failure of EGFR-TKI therapy: A multicenter retrospective study. J Transl Med (2019) 17(1):52. doi: 10.1186/s12967-019-1803-9

22. Liu L, Kalyani FS, Yang H, Zhou C, Xiong Y, Zhu S, et al. Prognosis and concurrent genomic alterations in patients with advanced NSCLC harboring MET amplification or MET exon 14 skipping mutation treated with MET inhibitor: A retrospective study. Front Oncol (2021) 11:649766. doi: 10.3389/fonc.2021.649766

23. Mi J, Huang Z, Zhang R, Zeng L, Xu Q, Yang H, et al. Molecular characterization and clinical outcomes in EGFR-mutant de novo MET-overexpressed advanced non-small-cell lung cancer. ESMO Open (2022) 7(1):100347. doi: 10.1016/j.esmoop.2021.100347

24. Song Z, Wang H, Yu Z, Lu P, Xu C, Chen G, et al. De novo MET amplification in Chinese patients with non–small-cell lung cancer and treatment efficacy with crizotinib: A multicenter retrospective study. Clin Lung Cancer (2019) 20(2):e171-e6. doi: 10.1016/j.cllc.2018.11.007

25. Tsui DCC, Lee JK, Frampton GM, Tolba K, Oxnard GR, Camidge DR, et al. Real-world (rw) analysis of quantitative MET copy number (CN) as a biomarker in NSCLC. J Clin Oncol (2022) 40(16_suppl):9123. doi: 10.1200/JCO.2022.40.16_suppl.9123

26. Bittoni M, Yang JC-H, Shih J-Y, Peled N, Smit EF, Camidge DR, et al. Real-world insights into patients with advanced NSCLC and MET alterations. Lung Cancer (2021) 159:96-106. doi: 10.1016/j.lungcan.2021.06.015

27. Kron A, Scheffler M, Heydt C, Ruge L, Schaepers C, Eisert A-K, et al. Genetic heterogeneity of MET-aberrant NSCLC and its impact on the outcome of immunotherapy. J Thoracic Oncol (2021) 16(4):572-82. doi: 10.1016/j.jtho.2020.11.017

28. Shalata W, Yakobson A, Weissmann S, Oscar E, Iraqi M, Kian W, et al. Crizotinib in MET exon 14-mutated or MET-amplified in advanced disease non-small cell lung cancer - A retrospective, single institution experience. Oncology (2022) 100(9):467-74. doi: 10.1159/000525188

29. Suryavanshi M, Shah A, Kumar D, Panigrahi MK, Metha A, Batra U. MET amplification and response to MET inhibitors in stage IV lung adenocarcinoma. Oncol Res Treat (2017) 40(4):198-202. doi: 10.1159/000457801

30. Tacar SY, Yilmaz M, Oz B, Tural D. Crizotinib for c-MET-amplified advanced NSCLC: a single-center experience. Tumori (2022) 108(3):258-62. doi: 10.1177/03008916211009303
